# Genetic Foundations of Nellore Traits: A Gene Prioritization and Functional Analyses of Genome-Wide Association Study Results

**DOI:** 10.3390/genes15091131

**Published:** 2024-08-27

**Authors:** Adebisi R. Ogunbawo, Henrique A. Mulim, Gabriel S. Campos, Hinayah R. Oliveira

**Affiliations:** Department of Animal Sciences, Purdue University, West Lafayette, IN 47907, USA; aogunbaw@purdue.edu (A.R.O.); hmulim@purdue.edu (H.A.M.);

**Keywords:** candidate genes, genomic region, over-representation, prioritization, QTL

## Abstract

The main goal of this study was to pinpoint functional candidate genes associated with multiple economically important traits in Nellore cattle. After quality control, 1830 genomic regions sourced from 52 scientific peer-reviewed publications were used in this study. From these, a total of 8569 positional candidate genes were annotated for reproduction, 11,195 for carcass, 5239 for growth, and 3483 for morphological traits, and used in an over-representation analysis. The significant genes (adjusted *p*-values < 0.05) identified in the over-representation analysis underwent prioritization analyses, and enrichment analysis of the prioritized over-represented candidate genes was performed. The prioritized candidate genes were *GFRA4*, *RFWD3*, *SERTAD2*, *KIZ*, *REM2*, and *ANKRD34B* for reproduction; *RFWD3*, *TMEM120A*, *MIEF2*, *FOXRED2*, *DUSP29*, *CARHSP1*, *OBI1*, *JOSD1*, *NOP58*, and *LOXL1-AS1* for the carcass; *ANKRD34B* and *JOSD1* for growth traits; and no genes were prioritized for morphological traits. The functional analysis pinpointed the following genes: *KIZ* (plays a crucial role in spindle organization, which is essential in forming a robust mitotic centrosome), *DUSP29* (involved in muscle cell differentiation), and *JOSD1* (involved in protein deubiquitination, thereby improving growth). The enrichment of the functional candidate genes identified in this study highlights that these genes play an important role in the expression of reproduction, carcass, and growth traits in Nellore cattle.

## 1. Introduction

Zebu cattle, also known as *Bos taurus indicus*, are prominently recognized by their pronounced hump, dewlap, heat tolerance, and tick resistance [1]. Over 8000 years ago, indicine cattle were known to have been domesticated in the Indus Valley [2]. Historically, Bos taurus indicus have been mainly selected for their ability to grow under harsh tropical environments and their capacity to maximize low-quality pasture; however, this resulted in a later age at puberty for Nellore cattle [3,4]. Nellore cattle are relevant in the global beef market and different breeding programs have been put in place to focus on genomic selection for the Nellore breed to accelerate the rate of genetic progress, reduce generational interval, and increase the accuracy of selection [5,6,7].

Genome-wide association studies (GWAS) have been used to identify genomic regions associated with important economic traits in Nellore cattle. GWAS uses the population linkage disequilibrium levels between markers and quantitative trait loci to identify genomic regions associated with a trait, thereby providing insights into their genetic architecture [8,9,10]. Examples of GWAS performed in Nellore cattle are Irano [11], who uncovered chromosomal regions associated with indicator traits of sexual precocity [11]; Silva [12], who identified regions that have a significant impact on stayability [12]; Carvalho [13], who identified genomic regions influencing growth traits [13]; and Reis [14], who identified the possible genomic regions and candidate genes associated with carcass traits [14]. 

Although GWAS are powerful tools for identifying genes and genetic variants associated with complex traits [10], the large number of GWAS has resulted in an extensive list of positional candidate genes for several important traits, with only a few validated through expression studies. This has created a gap in the comprehension of the genetic mechanism regulating these traits and made it difficult to determine which candidate genes are truly associated with the traits of interest. Oliveira [15] aimed to bridge this gap by fine-mapping genomic regions to pinpoint functional candidate genes specifically associated with reproductive traits in Nellore cattle [15]. By using these fine mapping techniques, the authors narrowed down the highly causal mutations and their associations with reproduction traits such as oocyte maturation and embryo development. Gene prioritization is a process through which the large list of candidate genes can be narrowed down and ranked to a smaller list of candidate genes, which could be potential causal variants for traits of interest [16]. Prioritizing candidate genes helps to optimize the fine-mapping process, as it ranks the long list of candidate genes before using fine-mapping. This is time-efficient and potentializes the validation process [17].

In the absence of appropriate gene prioritization analysis, a long list of candidate genes can hamper the identification of functional candidate genes [17]. Therefore, gene prioritization and functional analyses of GWAS results are paramount to pinpoint potential functional candidate genes associated with traits currently evaluated in Nellore cattle, which can then be further validated through gene expression studies and/or fine mapping. Recent studies have performed gene prioritization and functional analyses of GWAS results in beef and dairy cattle [18,19], but to the best of our knowledge, only two studies have specifically focused on the Nellore breed [20,21]. Nonetheless, Silva [20,21] has only investigated candidate genes associated with testicular hypoplasia and feet and leg malformations, as no other traits were included in their analysis. Moreover, no previous studies have identified the shared genetic background for a group of traits (such as growth, reproduction, and carcass traits), which may be paramount in identifying the key candidate genes impacting several traits together [20,21]. Consequently, the objectives of this study were as follows: (1) understand the main genetic factors controlling the main trait groups in Nellore cattle (i.e., reproduction, growth, carcass, and morphological traits); (2) pinpoint the functional candidate genes associated with these groups of traits within the Nellore cattle breed.

## 2. Materials and Methods

Approval from the Welfare and Animal Use Committee was not required for this study as the data were obtained from the literature. 

### 2.1. Data Gathering and Editing

This systematic review was guided by the standards of the Preferred Reporting Items for Systematic Review and Meta-Analysis (PRISMA) Statement [22,23]. A search for genomic regions associated with the Nellore breed was performed using a combination of keywords, as detailed in the Supplementary Materials Table S1. The keywords were created using the following criteria: terms related to economically important traits (e.g., reproduction, morphological, carcass, growth, scrotal circumference, and age at puberty); type of analysis (e.g., GWAS, candidate genes, genome-wide association, and genome-wide association studies); and specific cattle breed (e.g., Nellore cattle). The keyword combination was used to search the Animal QTL database [24], National Center for Biotechnology Information [25], Web of Science (www.webofscience.com/; accessed on 15 January 2024), and Elicit (Elicit: The AI Research Assistant; www.elicit.com; accessed on 20 November 2023). Covidence (www.covidence.org/) was used to extract and screen the papers. Additionally, the GALLO package [26] available in the R software 4.3.1 [27] was also used to search for papers by focusing on the gene annotation database recorded on the Nellore breed. 

**Figure 1:**
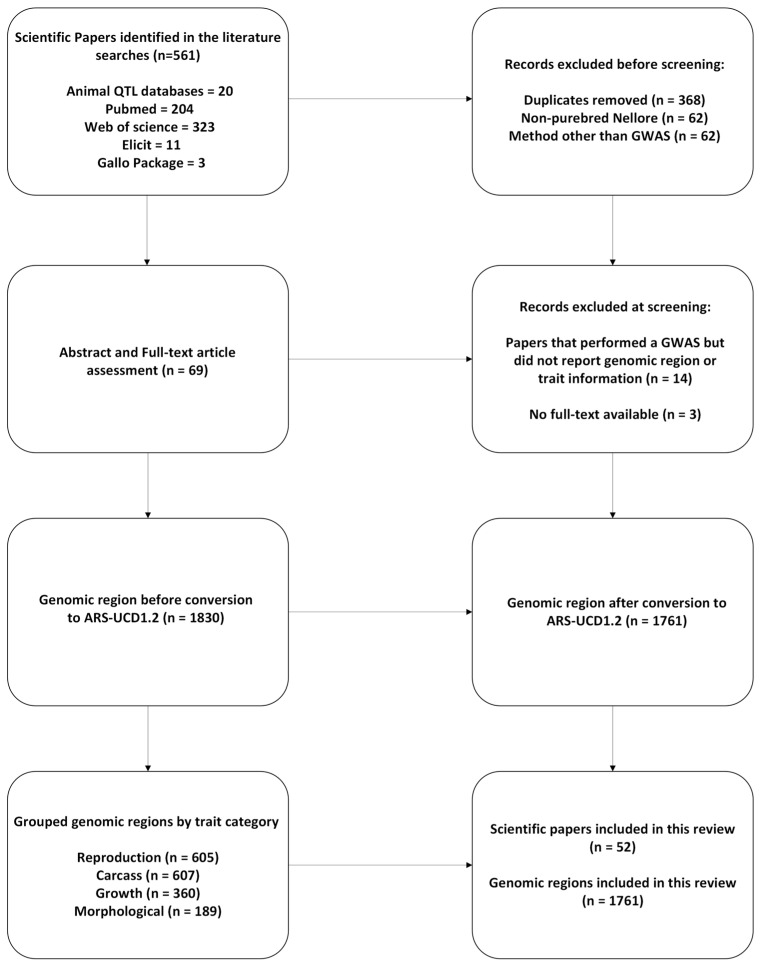
Flow diagram of the search strategy used to select scientific papers in genome-wide association studies in Nellore cattle. Adapted from the preferred reporting items for systematic reviews (PRISMA [22,23]).

A total of 561 scientific papers were first identified, genomic regions and single nucleotide polymorphism (SNP) markers reported as significant in association with target traits were recorded, and a quality control procedure was performed. The first control step consisted of removing duplicate papers (*N* = 368) and conference proceeding abstracts that were not full-text publications (*N* = 3); subsequently, papers with undefined traits and/or papers that did not report the genomic positions were also removed (*N* = 14). Finally, papers that did not use purebred Nellore and/or perform genome-wide association analysis were also removed (*N* = 62). A scheme of the quality control steps used in this study is shown in Figure 1. In the end, 1830 genomic regions reported in 52 scientific papers were available for analysis.

When studies did not provide specific genomic coordinates for SNP markers and candidate genes, we retrieved them using the QTL ID, SNP ID, Ensembl gene ID, or gene name information provided in the literature, which were converted using the QTL database (QTLdb) [24], SNPchimp [28], and/or Ensembl genome browser [29]. The physical position of all genome coordinates was updated to the latest reference genome ARS-UCD1.2 [30] using Lift Genome Annotations [31], and the center of the updated genomic coordinates was used for gene annotation. Positional candidate genes were annotated within a 1MB interval (with 500 Kb upstream and 500 Kb downstream) using the GALLO package [26]. The final complete dataset used in this study is available in the Supplementary Materials Table S2. 

### 2.2. Trait Groups

The recorded traits were identified and categorized into four groups, each comprising a subgroup or indicator trait to further understand the genetic background of the traits. The groups are defined as follows:

1. Reproduction traits: scrotal circumference, age at puberty, early pregnancy, early puberty, precocity, age at first calving, heifer rebreeding, number of calving, testicular hypoplasia, gestation length, preweaning calf mortality rate, antral follicle count, stayability, anti-Müllerian hormone, gestation length, calving interval, calving ease, and post-natal mortality.
  1.a. Subgroup: Sexual precocity
    Included traits: scrotal circumference, early pregnancy, early puberty, age at first calving, antral follicle count, calving interval, and calving ease.
2. Carcass traits: backfat thickness, rib eye area, rump fat thickness, hot carcass weight, subcutaneous fat thickness, intramuscular fat content, longissimus muscle area, marbling, tenderness, shear force tenderness, and meat color.
  2.a. Subgroup: Meat quality
    Included traits: marbling, tenderness, shear force tenderness, and meat color.
3. Growth-related traits: residual body weight gain, average daily gain, accumulated productivity, birth weight, weaning weight, yearling weight, adult cow weight, weight gain from birth to weaning, weight gain from weaning to yearling, yearling height, residual feed intake, dry matter intake, feed efficiency, and feed conversion ratio.
  3.a. Subgroup: Efficiency
    Included traits: residual feed intake, dry matter intake, feed efficiency, and feed conversion ratio.
4. Morphological traits: body conformation, muscularity, precocity, feet and leg deformation, and feet and leg conformation.
  4.a. Subgroup: Visual scores
    Included traits: conformation, precocity, and muscling.

A final summary of the number of studies, genomic regions, and positional candidate genes retrieved per trait group is shown in Table 1.

### 2.3. Over-Representation and Prioritization Analyses 

Positional candidate genes annotated for each trait group were used in an over-representation analysis (ORA). The ORA was performed using the Medical Subject Headings (MeSH) vocabulary to retrieve annotations that appear more frequently in a selected gene group relative to their occurrence by chance [32]. The statistical significance (*p*-value) of ORA was assessed using the hypergeometric test [33]:

$$p=\sum_{i=k}^{min(M,n)}\frac{\left(\begin{array}{c} M\\ i \end{array}\right)\left(\begin{array}{c} N-M\\ n-i \end{array}\right)}{\left(\begin{array}{c} N\\ n \end{array}\right)},$$

where N is the total number of genes that were analyzed for each trait (reference genes), M is the total number of selected genes, n is the total number of genes in the MeSH term under study, k is the number of selected genes that belong to the MeSH term under study, and $\left(\begin{array}{c} y\\ x \end{array}\right)=\frac{y!}{x!}\left(y-x\right)!$ is the binomial coefficient where y is the total number of genes analyzed and x is the number of genes selected from the set of y genes [34]. To increase the power of the test, all traits within the same trait group were analyzed together in the ORA. The ORA was performed using the MESHR package [35,36]. Significant genes (i.e., adjusted *p*-value < 0.05) from the ORA were then considered “test genes” for the prioritization analysis.

The “guilt by association” prioritization analysis was performed using the software GUILDify v2.0 and ToppGene [18,37,38,39]. The “guilt by association” principle suggests that genes with similar biological functions tend to be associated, thereby allowing the statistical inference of a gene’s function based on the association and prior knowledge of other genes [18,37,38]. First, a “trained list” of the top 200 ranked genes with a guild score > 0.6, which is a likelihood score to assess the relevance of associated preselected relevant trait keywords, was obtained from the GUILDify v2.0 Web Server [19,40]. The selected keywords are as follows:

- Reproduction and sexual precocity traits: “Fertility”, “implantation”, “preimplantation”, “endometrium”, “embryonic development”, “primordial follicles”, “uterus”, “luteal”, “gestation”, “embryo”, “ovulation”, “estrogen”, “estradiol”, “endocrine hormone”, “gamete, mammary glands”, “lactation”, “pregnancy”, “oocyte”, “fetus”, “zygote”, “ovary”, “amniotic”, “cervical mucosa follicle”, “Gonad”, “antral follicles”, “pre-eclampsia”, “placenta”, “testes”, “sperm”, “scrotal circumference”, “scrotal, testicular”, “testis”, “semen”, “spermatozoa”, “spermatogenesis”, “testicular hypoplasia”, and “hypogonadism”.
- Carcass and meat quality traits: “Longissimus muscle”, “Backfat thickness”, “Myosin”, “Actin”, “Fibroblast”, “Tissue deposition”, “Fat deposition”, “Muscle, Subcutaneous fat”, “Biceps femoris”, “Carcass weight”, and “Skeletal muscle”.
- Morphological and visual score traits: “body development”, “muscle mass”, “skeletal muscle”, “subcutaneous fat”, “conformational structure”, “adipose tissue”, “body depth”, “feet and leg”, “foot angle”, “tendon”, “joints”, “mobility aplomb”, “osteogenic differentiation”, “articular cartilage”, and “fat deposition”.
- Growth-related and efficiency traits: “body weight”, “birth weight”, “adult weight”, “weight gain”, “body size”, “average daily gain”, “body weight gain”, “daily gain”, “stature”, “growth”, “adipose tissue”, “dry matter intake”, “metabolism”, “maternal behavior”, “maternal ability”, “feed conversion”, “feed efficiency”, “dry matter”, “milk composition”, and “muscle development”.

The GUILDify software uses a Biologic Interaction and Network Analysis (BIANA) knowledge database to query gene products associated with keywords and rank the genes in this analysis [40]. This software uses a selected list of genes and a species-specific (*Homo sapiens*) protein interaction network and applies graph theory algorithms to prioritize genes [19,40]. A network-based prioritization algorithm “Netscore” with three repetitions and two iterations was used to prioritize gene associations and protein–phenotype interactions [38,39]. 

The ToppGene software was used to perform an annotation-based prioritization analysis through a fuzzy-based multivariate approach [37]. The functional information shared between the “trained” gene list and the “test” gene list was used to perform the multivariate analysis [37]. This functional information was retrieved from the following sources: Gene Ontology (GO) terms for molecular function (MF), biological process (BP), and cellular component (CC); human and mouse phenotypes; metabolic pathways; PubMed publications; and diseases. Using a statistical meta-analysis, the *p*-values obtained in a random sampling of 5000 genes from the whole genome for each annotation information were combined in an overall *p*-value. Gene ranking was performed by applying the PPIN-based candidate gene prioritization and the K-Step Markov method [37,40]. A total of 200 training genes were used for all trait groups and subgroups, and the number of test genes was 379 for reproduction, 277 for sexual precocity, 2016 for carcass, 835 for meat quality, 187 for growth traits, 224 for efficiency, 91 for morphological, and 30 for visual score traits. A false discovery rate (FDR) of 5% multiple testing correction (*p*-value ≤ 10^−3^) was used, and the significant prioritized genes were used in further analysis [18,40].

### 2.4. Functional Analysis

Enrichment analysis of the significant prioritized genes shown in the Supplementary Materials Table S6, was performed using the Clusterprofiler to investigate their roles in the BP, MF, and CC [41]. The Bioconductor annotation package “org.Hs.eg.db” was imported for the genome-wide annotation using mapping Entrez Gene identifiers for humans, as the prioritization analysis also used a *Homo sapiens* interaction network [41,42]. Significant prioritized genes have the same functional profile as the genes on the “trained” list [37]. This approach has been used in several studies with cattle [18,19,20,42]. 

### 2.5. Venn Diagrams and Gene Network Integration

The official gene names of the prioritized genes were used to integrate the available information and create the Venn diagrams. The Venn diagram tool available at www.bioinformatics.psb.ugent.be/webtools/Venn/ was used to calculate intersections between the gene lists, thereby depicting what genes overlap or are unique to each trait group. To investigate possible interactions among the prioritized genes in each categorized trait, the GeneMANIA web tool (implemented as a plug-in on the Cystoscape platform) was used [43]. This network analysis searches for related genes on publicly available biological datasets and classifies the link in the network based on their relationships, such as co-expressions, physical interactions, genetic interactions, shared protein domains, co-localization, and pathways. These analyses give a better understanding of the genetic architecture of the complex polygenic traits, in order to show functions associated with genes in the network and their false discovery rate and coverage [44].

## 3. Results and Discussion

After quality control, a total of 52 articles were used in the analyses, as summarized in Figure 1. Of these, 23 articles focused on reproduction traits, 14 articles on carcass traits, 12 on growth traits, and five articles on morphological traits.

### 3.1. Gene Annotation and Prioritization Analysis

The 52 articles selected contained a total of 1761 unique genomic regions, 605 genomic regions associated with reproduction, 387 with sexual precocity, 607 with carcass traits, 294 with meat quality, 360 with growth, 216 with efficiency, 189 with morphological traits, and 132 with visual scores. These genomic regions were annotated to identify candidate genes resulting in 8569 positional candidate genes for reproduction traits, 5412 for sexual precocity, 11,195 for carcass traits, 6646 for meat quality, 5239 for growth traits, 3319 for efficiency traits, 3483 for morphological traits, and 2114 for visual score traits. To mitigate potential biases, particularly those arising from selective reporting of candidate genes, we focused on genomic regions rather than individual SNPs or genes. By performing our own gene annotation within these regions, we ensured that all relevant positional candidate genes were consistently reported, regardless of the original study’s methodology. Interestingly, only a few genomic regions were similar across different studies for the same trait. For instance, two similar genomic regions were found across four studies for the age at first calving; one similar genomic region was found across two studies for early pregnancy; three similar genomic regions were found across five studies for backfat thickness; one similar genomic region was found across two studies for the rib eye area, rump fat thickness, hot carcass weight, and longissimus muscle area traits; one similar genomic region was found across two studies for the average daily gain and birth weight; and one similar genomic regions was found across two studies for precocity. 

The candidate genes annotated for each trait group and subgroup were used in the over-representation analysis, where the numbers of significant genes identified in each category were 379 for reproduction traits, 277 for sexual precocity, 2016 for carcass, 835 for meat quality, 187 for growth, 224 for efficiency, 91 for morphological traits, and 30 for visual scores. These over-represented significant genes were considered “test genes” for the prioritization analysis. For the prioritization analysis, a list of 200 ranked genes (Supplementary Materials Table S3), derived from keywords related to the traits of interest, was used. This annotation-based prioritization analysis resulted in six prioritized genes for reproduction traits, two for sexual precocity, 10 for carcass, two for meat quality, two for growth, two for efficiency, and no prioritized genes for morphological and visual score traits. The list of prioritized genes for each trait group is included in the Supplementary Materials Table S4.

The variation in the number of candidate genes prioritized across trait groups may be attributed to the differential focus of research efforts on specific traits in Nellore cattle, in addition to the different genetic backgrounds of the traits. For instance, out of 52 total articles used in this study, 23 focused on reproduction traits, 14 on carcass traits, 12 on growth traits, and only five on morphological traits. In this context, the relatively few studies on growth and morphological traits likely contributed to the identification of only two prioritized genes for growth and none for morphological traits. Consequently, there is a pressing need for more GWAS that focus on understanding the genomic regions associated with growth and morphological traits in Nellore cattle.

### 3.2. Enrichment and Gene Integration Analysis of the Functional Candidate Genes Identified within Groups

#### 3.2.1. Reproduction and Sexual Precocity Traits

The six candidate genes prioritized for reproductive traits were *GFRA4*, *RFWD3*, *SERTAD2*, *KIZ*, *REM2*, and *ANKRD34B*. Among these, the *ANKRD34B* gene is common to both reproductive traits and sexual precocity, while the *ACSM4* gene is uniquely prioritized for the sexual precocity trait. Gene functions of the six prioritized genes for reproductive traits and the two for sexual precocity traits were identified using GeneMANIA. The results indicated significant interactions among the genes associated with both traits: 80.30% and 77.64% physical interactions, suggesting that the genes are linked through protein–protein interactions; 9.48% and 5.37% predicted interactions, indicating that the genes likely participate in the same pathway reactions; 4.28% and 2.87% genetic interactions, showing that the genes are functionally associated, with the effect of one gene being modified by another; 3.72% and 8.01% co-expressions, demonstrating similar patterns across gene expression studies; 0.74% and 3.63% co-localizations, indicating that the genes are expressed in the same tissues; and 0.37% and 0.60% shared protein domains, showing common protein domain data among the genes.

**Figure 2:**
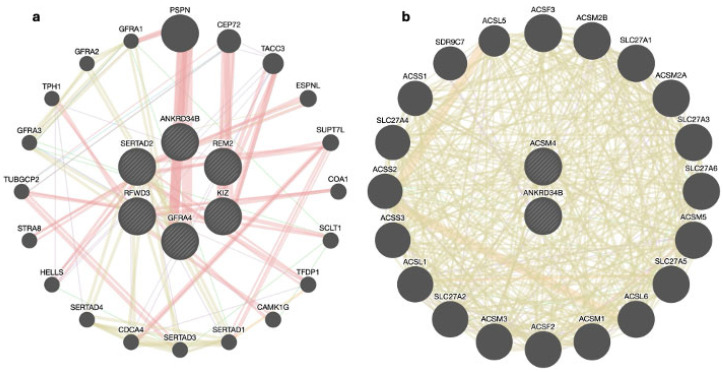
Interactions between prioritized genes among reproduction (**a**) and sexual precocity (**b**) traits.

Related genes, such as *CEP72*, *TUBGCP2*, and *ESPNL* (Figure 2a), and *ACSM2A* and *SLC27A1* (Figure 2b), are associated with the prioritized genes through various biological functions. These genes are involved in the organization of the mitotic cell cycle, binding of actin filaments, and expression of reproductive traits such as the bipolar spindle, endometrium, and ovary [45,46,47]. Additionally, they encode mitochondrial acyl-coenzyme A and other fatty acids needed in the pathway [48,49].

The *KIZ* gene plays a crucial role in spindle organization in Nellore cattle, which is essential for forming a robust mitotic centrosome architecture capable of withstanding the forces exerted on the centrosome during spindle formation [50]. The *SERTAD2* gene is involved in the regulation of cell growth, which is important in the reproduction process. For instance, Zhang et al. [51] observed that patients with lower levels of *lnc-SERTAD2-3*, a member of the *SERTAD* family, had an unfavorable prognosis for osteosarcoma [51]. In contrast, patients with higher levels of *lnc-SERTAD2-3* experienced inhibited cell growth and spreading, confirming the role of the *SERTAD2* gene in cell growth regulation. Additionally, Darwish [52] found that inhibiting *SERTAD3*, another member of the *SERTAD* gene family, resulted in a two- to four-fold reduction in cell growth rate [52].

The *RFWD3* gene is associated with DNA recombination, DNA replication, DNA damage checkpoint signaling, and mitotic DNA damage regulation of the cell cycle phase. Fu et al. [53] investigated the role of the *RFWD3–Mdm2* ubiquitin ligase complex in regulating p53 protein stability following DNA damage [53]. They identified that *RFWD3* forms a complex with p53 to synergistically facilitate its ubiquitination, thereby playing a crucial role in cellular protection by regulating the response to DNA damage. The *REM2* gene is associated with guanyl nucleotide (GTP) binding, a protein crucial for the development and maturation of reproductive cells. Casalotti et al. [54] investigated the expression of GTP-binding proteins during the development of rat testes and discovered that the cellular expression of G proteins is temporally linked to testicular development in rats [54]. 

The *ACSM4* gene plays a significant role in the mitochondrial matrix, which is crucial for cellular energy metabolism [55]. The mitochondrial matrix houses the citric acid cycle and enzymes such as C-acyltransferase and CoA-ligase, which are important for improving steroidogenesis, a process essential for the secretion of steroid hormones such as testosterone [56,57].

#### 3.2.2. Carcass and Meat Quality Traits

The ten candidate genes prioritized for carcass traits were *RFWD3*, *TMEM120A*, *MIEF2*, *FOXRED2*, *DUSP29*, *CARHSP1*, *OBI1*, *JOSD1*, *NOP58*, and *LOXL1-AS1*. Among these, *CARHSP1* and *LOXL1-AS1* were also prioritized for meat quality traits. However, the genes *DUSP29* and *LOXL1-AS1* were not recognized in the GeneMANIA network. The *LOXL1-AS1* gene is a non-protein-coding antisense RNA gene, and *DUSP29* was likely not found in GeneMANIA due to the lack of existing data sources in the interaction database.

**Figure 3:**
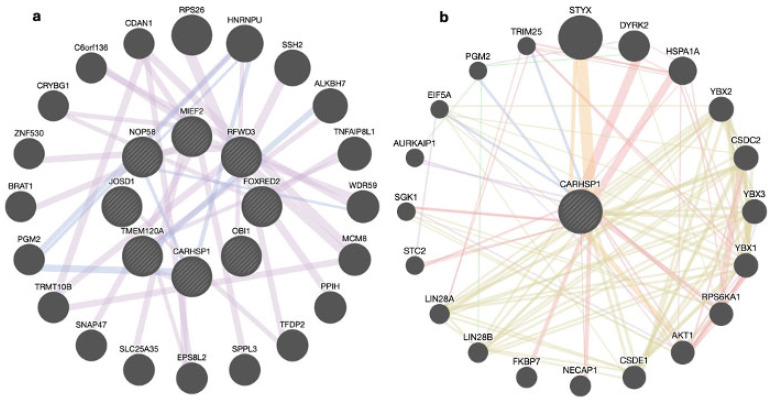
Interactions between prioritized genes among carcass (**a**) and meat quality (**b**) traits.

The eight recognized prioritized genes for carcass traits as seen in Figure 3a (i.e., *RFWD3*, *TMEM120A*, *MIEF2*, *FOXRED2*, *OBI1*, *JOSD1*, and *NOP58*) showed 71.22% co-expressions, indicating similar expression levels across conditions, and 28.78% co-localization, indicating that these genes are expressed in the same tissues associated with carcass traits. The one prioritized gene for meat quality traits as seen in Figure 3b, *CARHSP1*, showed 77.64% physical interactions, suggesting that the genes are linked through protein–protein interactions; 8.01% co-expressions, suggesting similar expression levels across conditions; 5.37% predicted interactions and 1.88% pathway participation, suggesting that the genes likely participate in the same pathway reactions; 3.63% co-localization, indicating expression in the same tissues; 2.87% genetic interactions, showing functional association with the effect of one gene being modified by another; and 0.60% shared protein domains, indicating common protein domain data among the genes.

Related genes such as *ALKBH7*, *CDAN1*, *DYRK2*, *HSPA1A*, and *SNAP47* are involved in stabilizing existing proteins, cellular response to DNA damage, and biological functions related to carcass or meat quality, such as the nuclear envelope, which surrounds the nucleus, and intracellular membrane fusion [58,59,60,61,62].

The *OBI1* gene is involved in the regulation of DNA replication and protein autoubiquitination and/or deubiquitination [63]. Nassar et al. [64] studied the mechanism behind *OBI1* function using Xenopus laevis eggs and confirmed that the *OBI1* gene encodes an E3 ubiquitin ligase, a protein essential for DNA replication in the genome. This gene plays a critical role in regulating DNA replication and maintaining cellular homeostasis through protein autoubiquitination or deubiquitination processes, which are important for ensuring good meat quality [64]. 

The *TMEM120A* gene is associated with sensory perception, which is important in the detection of mechanical external stimuli and fat cell differentiation [65]. Batrakou et al. [66] investigated the roles of two nuclear envelope transmembrane proteins, *TMEM120A* and *TMEM120B*, in adipocyte differentiation and metabolism. They confirmed that the nuclear envelope transmembrane protein *TMEM120A* is preferentially expressed in fat and is required for adipocyte differentiation and metabolism [66].

The *DUSP29* gene is involved in protein dephosphorylation, which activates or inactivates enzymes and is also involved in muscle cell differentiation, an important characteristic of meat quality [67]. Cooper et al. [68] determined how *DUSP29* is transcriptionally regulated in skeletal muscle and confirmed the importance of *DUSP29* in muscle cell differentiation, providing insight into molecular and cellular mechanisms for skeletal muscle atrophy [68].

The *JOSD1* gene is involved in protein modification and deubiquitination, reversing the ubiquitination process and preventing protein degradation, thereby improving meat quality [69]. Seki et al. [70] provided additional insights into the properties of the Josephin domain (*JOSD*) and confirmed that *JOSD1* is activated by ubiquitination and helps regulate membrane dynamics [70].

The two candidate genes prioritized for meat quality traits, which were also prioritized for carcass traits, are *LOXL1-AS1* and *CARHSP1*. *LOXL1-AS1* is a non-protein-coding antisense RNA [71], while *CARHSP1* is involved in RNA binding to the 3′-UTR ends of histones [72], a process recently linked to adipose tissue accumulation in pigs [73]. Kociucka et al. [73] investigated the association between histone modifications and metabolism in pig adipose tissues, helping to understand adipose tissue development, which is essential for improving meat quality in pigs [73].

#### 3.2.3. Growth-Related and Efficiency Traits

**Figure 4:**
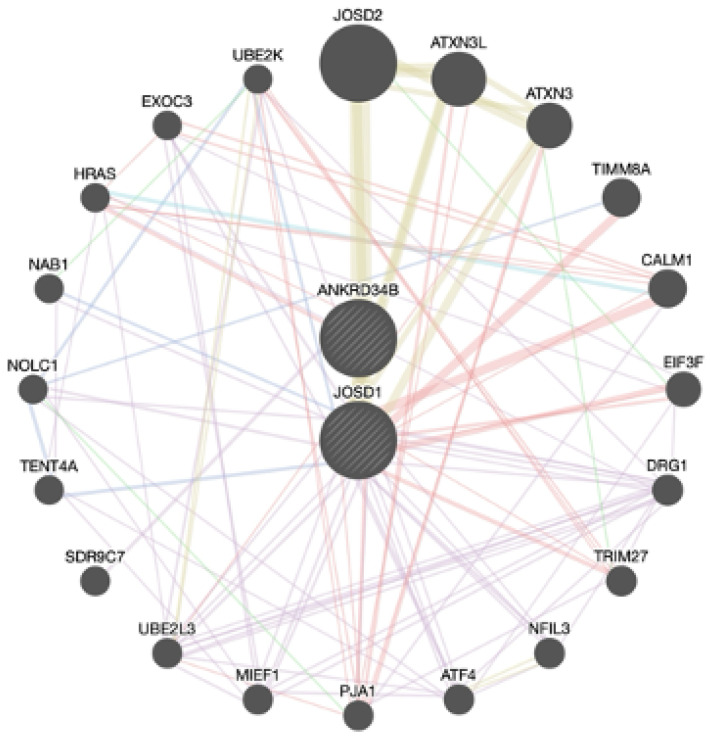
Interactions between prioritized genes among growth and efficiency traits.

The candidate genes prioritized for growth-related and efficiency traits were the same, indicating that the genes identified are key genes regulating the expression of both growth-related and efficiency traits. The two prioritized genes for these trait groups are *ANKRD34B* and *JOSD1*, and their interactions with other genes are shown in Figure 4. The following interactions were observed: 77.64% physical interactions, 8.01% co-expressions, 5.37% predicted interactions, 2.87% genetic interactions, 1.88% pathways, and 0.60% shared protein domains. Related genes, such as *TIMM8A*, *EIF3F*, *PJA1*, and *TENT4A,* are involved in the insertion of hydrophobic membrane proteins in the mitochondrial membrane and associated with enabling protein deubiquitination and DNA repair [74,75].

In addition to its role in reproduction processes [76], such as germ and pole plasm, *ANKRD34B* is also associated with ribonucleoprotein granules, which are complexes of RNA and RNA-binding proteins that play essential roles in regulating cell growth and development [77]. To address limitations related to ribonucleoproteins, Tartaglia [77] explored the crucial role of protein–RNA interactions in cellular regulation and the gaps in understanding how ribonucleoprotein complexes assemble and function. Their study found that these ribonucleoproteins are essential for various cellular processes, including growth [77]. Regarding the *JOSD1* gene, this gene is involved in protein deubiquitination, which reverses the ubiquitination process and prevents protein degradation, thereby improving growth [69]. This gene also plays a significant role in cellular processes, as it participates in the localization of plasma membranes, affecting endocytosis [70].

#### 3.2.4. Morphological and Visual Score Traits

No candidate genes were prioritized for morphological and visual score traits; therefore, no gene was recognized by the network integration algorithm. The limited number of studies on morphological and visual score traits likely contributed to identifying no prioritized genes for both trait groups, as only five and three scientific publications were found for morphological and visual score traits, respectively. 

### 3.3. Genes Shared among Groups 

#### 3.3.1. Over-Represented Genes 

**Figure 5:**
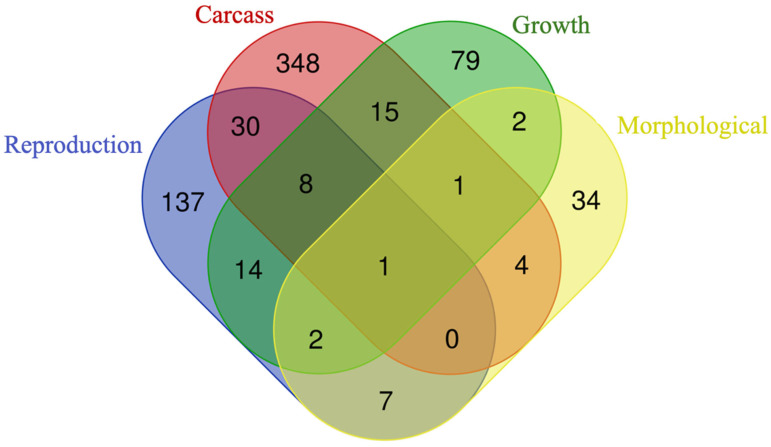
Venn diagram displaying the unique and shared over-represented genes among reproduction, carcass, growth, and morphological traits.

After assessing the over-representation of positional candidate genes, the intersections between gene lists were evaluated (Figure 5). Of the genes analyzed, 137 out of 379 were unique to reproduction traits, 348 out of 2016 were unique to carcass traits, 79 out of 187 were unique to growth traits, and 34 out of 91 were unique to morphological traits.

In terms of shared genes, one gene was common among all four trait groups. Eight genes were common to reproduction, carcass, and growth traits, while two genes were common to reproduction, growth, and morphological traits. Only one gene was shared among carcass, growth, and morphological traits. Additionally, 30 genes were shared between reproduction and carcass traits, 14 genes between reproduction and growth traits, and seven genes between reproduction and morphological traits. The carcass and growth traits shared 15 genes, the carcass and morphological traits shared four genes, and the growth and morphological traits shared two genes. The complete list of over-represented genes identified per trait group and subgroup is included in the Supplementary Materials Table S5. 

#### 3.3.2. Prioritized Genes

**Figure 6:**
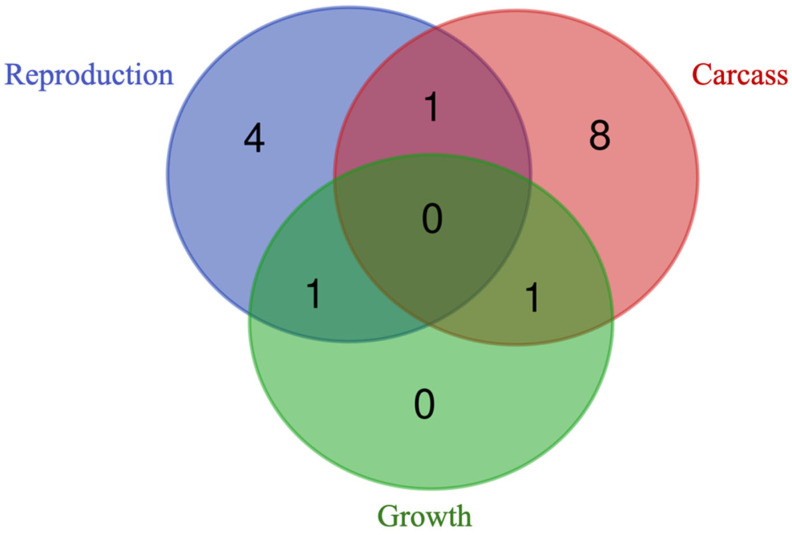
Venn diagram displaying the unique and shared prioritized genes among reproduction, carcass, and growth traits. No prioritized genes were found for the morphological traits.

Four genes (i.e., *GFRA4*, *KIZ*, *REM2*, and *SERTAD2*) were found to be unique to the reproduction traits, while eight genes (i.e., *OBI1*, *NOP58*, *DUSP29*, *TMEM120A*, *LOXL1-AS1*, *MIEF2*, *CARHSP1*, and *FOXRED2*) were unique to the carcass traits. Notably, no genes were found to be unique to the growth traits (Figure 6). The reproduction and carcass traits shared the gene *RFWD3*, the reproduction and growth traits shared the gene *ANKRD34B*, and the carcass and growth traits shared the gene *JOSD1*.

### 3.4. Genes Shared between Groups and Subgroups

**Figure 7:**
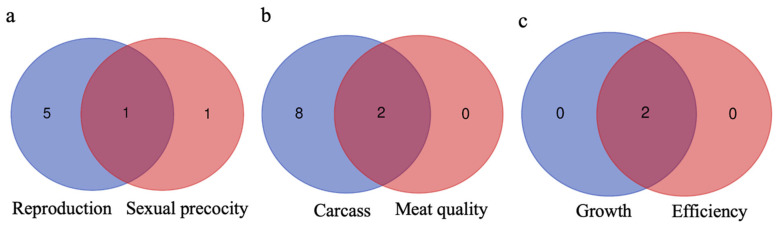
Venn diagram displaying the unique and shared prioritized genes between trait groups and their respective subgroups. Groups and subgroups are (**a**) reproduction and sexual precocity; (**b**) carcass and meat quality; and (**c**) growth and efficiency. No prioritized genes were found for the morphological traits.

The intersections of genes between trait groups and their respective subgroups are shown in Figure 7. Six prioritized genes—*GFRA4*, *RFWD3*, *SERTAD2*, *KIZ*, *REM2*, and *ANKRD34B*—are unique to reproduction traits. Among these, *ANKRD34B* is also common to both reproduction and sexual precocity traits, with *ACSM4* being uniquely prioritized for sexual precocity.

The intersection between carcass and meat quality traits revealed two commonly prioritized genes: *LOXL1-AS1* and *CARHSP1*. Eight prioritized genes—*RFWD3*, *DUSP29*, *TMEM120A*, *CARHSP1*, *OBI1*, *LOXL1-AS1*, *FOXRED2*, and *NOP58*—are unique to carcass traits, with no genes unique to meat quality traits. In the intersection between growth and efficiency traits, two prioritized genes—*ANKRD34B* and *JOSD1*—are shared, with no genes unique to either trait. No prioritized genes were identified for morphological and visual score traits.

The Venn diagram was used to calculate intersections between gene lists and illustrate the overlap of candidate genes among reproduction, carcass, and growth traits in Nellore cattle. By examining these intersections, we identify shared genes that may be pleiotropic, meaning that one gene affects the expression of multiple phenotypes [78].

The *RFWD3* gene, common to both reproduction and carcass traits, plays a role in DNA repair, DNA replication, DNA recombination, and ubiquitination [79]. Efficient DNA repair mechanisms are crucial for maintaining genetic integrity during gametogenesis and embryonic development—a key aspect of reproduction [80]. Inano et al. [81] studied *RFWD3*’s role as an E3 ubiquitin ligase in the DNA damage response. Their research revealed that *RFWD3* facilitates homologous recombination and proper DNA repair by ubiquitinating and removing RPA and RAD51 proteins, preventing interference with the repair process [81]. The gene’s involvement in DNA repair, replication, and recombination contributes to meat quality and yield, highlighting its dual role in both reproduction and carcass traits [79,80,81].

The *ANKRD34B* gene is shared between reproduction and growth traits. Known for its association with ribonucleoprotein granules—a complex of RNA and RNA-binding proteins—*ANKRD34B* plays essential roles in regulating cellular processes such as cell regulation, growth, and development [76]. This may influence gametogenesis or early embryo development [77]. Further research is needed to understand how *ANKRD34B* influences the reproduction process. The *JOSD1* gene is common to both carcass and growth traits. This gene, involved in protein modification and deubiquitination, helps prevent protein degradation, thereby improving growth-related and meat quality traits. *JOSD1*’s role in maintaining cellular protein balance through deubiquitination is crucial for muscle quality and growth regulation, impacting muscle composition and carcass quality [70].

## 4. Limitations of This Study and Future Directions 

Different statistical models were used in the original GWAS analyses, and we relied on the assumption that each study employed appropriate modeling, accounting for all relevant fixed and random effects influencing the traits of interest. This assumption is a standard practice in meta-analysis and systematic reviews (e.g., [18,19,82]). While it is recognized that incorrect models or small sample sizes could lead to an increased risk of false positives or negatives, our over-representation and gene prioritization analyses were designed to detect and mitigate the impact of spurious associations. Specifically, these analyses help identify patterns of gene enrichment that are consistent across multiple studies, reducing the likelihood that any single study’s methodological limitations would disproportionately affect our overall findings. However, future studies should compare novel alternatives to account for the reliability of the studies used. 

A primary limitation of this study was the absence of a cattle-specific database in the GUILDify software, which required the use of a human (*Homo sapiens*) protein interaction network for gene prioritization. Although cattle share approximately 80% of human orthologs [83,84], using a human database instead of a cattle-specific one may have affected the results. To address this limitation, future efforts should focus on developing and integrating a comprehensive cattle-specific protein interaction database into GUILDify. Additionally, enhancing cross-species mapping algorithms and improving cattle genome annotation could ensure that the genetic and protein interaction data used in studies are more accurate and relevant to cattle, thereby mitigating the impact of using human databases. Furthermore, GUILDify employs multiple algorithms (e.g., NetScore, NetZcore, and NetShort) to prioritize genes based on protein–protein interactions [38]. However, this study utilized the default NetScore algorithm with a repetition of three and an iteration of two, as recommended by the software developer. Future studies could compare the performance of multiple algorithms to determine their impact on the results. Regardless, it is important to note that the GUILDify software ranks these genes based on network topology, while the ToppGene software ranks the candidate genes based on functional similarity. These differences may help reduce the potential bias from the algorithm used for GUILDify in this study.

Moreover, this study was limited to GWAS performed using SNP panels, which were more widely available for the Nellore breed. As a result, some significant genomic markers associated with traits of interest may have been overlooked. To address this limitation, future studies should incorporate a broader range of molecular technologies, including whole-genome sequencing, copy number variation analysis, transcriptomics, and epigenomics. Integrating data from these sources could provide a more comprehensive understanding of the genetic architecture of these traits and ensure that all relevant genetic markers are identified.

## 5. Conclusions

The candidate genes identified in this study enhance our understanding of the genetic architecture and key genetic mechanisms controlling reproduction, carcass, growth, and morphological traits in the Nellore breed. The observed overlap of genes among traits underscores their complexity and indicates that many of these genes have cross-phenotype effects, where a single candidate gene is associated with multiple traits and shares common genetic pathways. The functional candidate genes identified in this study can serve as targets for gene expression and fine mapping studies, which are crucial for validating these findings and further elucidating their roles in trait regulation.

## Figures and Tables

**Table 1:** Summary of the number of studies, genomic regions, and positional candidate genes retrieved per trait group.

^1^ Trait Groups	Studies	Genomic Regions	Candidate Genes
Reproduction	23	605	8569
Sexual precocity	19	387	5412
Carcass	14	607	11,195
Meat quality	8	294	6646
Growth	12	360	5239
Efficiency	7	217	3319
Morphological	5	189	3483
Visual scores	3	132	2114

^1^ The trait groups are as follows: (1) Reproduction traits: scrotal circumference, age at puberty, early pregnancy, early puberty, precocity, age at first calving, heifer rebreeding, number of calving, testicular hypoplasia, gestation length, preweaning calf mortality rate, antral follicle count, stayability, anti-Müllerian hormone, gestation length, calving interval, calving ease, post-natal mortality. (1.a) Sexual precocity subgroup: scrotal circumference, early pregnancy, early puberty, age at first calving, antral follicle count, calving interval, and calving ease. (2) Carcass traits: backfat thickness, rib eye area, rump fat thickness, hot carcass weight, subcutaneous fat thickness, intramuscular fat content, longissimus muscle area, marbling, tenderness, shear force tenderness, and meat color. (2.a) Meat quality subgroup: marbling, tenderness, shear force tenderness, and meat color. (3) Growth-related traits: residual body weight gain, average daily gain, accumulated productivity, birth weight, weaning weight, yearling weight, adult cow weight, weight gain from birth to weaning, weight gain from weaning to yearling, yearling height, residual feed intake, dry matter intake, feed efficiency, and feed conversion ratio. (3.a) Efficiency subgroup: Residual feed intake, dry matter intake, feed efficiency, and feed conversion ratio. (4) Morphological traits: body conformation, muscularity, precocity, feet and leg deformation, and feet and leg conformation. (4.a) Visual scores subgroup: conformation, precocity, and muscling.

## Data Availability

All data used in this study are included in the main text and the Supplementary Materials (Tables S1–S6).

## Supplementary Materials

The following supporting information can be downloaded at https://www.mdpi.com/article/10.3390/genes15091131/s1, Table S1: Keyword combination used to search database; Table S2: Final scientific publication used in this study, which includes genomic regions associated with trait group; Table S3: Training genes data for each grouped trait. Each worksheet shows the grouped trait, the keywords used in GUILDify, gene identification of each gene (Entrez Gene ID), and the guild score of each gene, which shows how associated the gene is to the keyword; Table S4: Prioritized gene list for each trait group. Each worksheet represents the prioritized gene in each trait group along with gene identification (i.e., GeneSymbol and GeneId), average score, and overall *p*-value. Table S5: Significant genes identified in the over-representation analysis. Each worksheet represents the significant genes after over-representation analyses (also called test genes) in each trait group and subgroup along with gene identification (i.e., Entrez Gene ID). Table S6: Enrichment analysis result for the biological process, cellular component, and morphological function of each trait group along with Gene Ontology term (ID), description of enriched term corresponding to ID, GeneRatio of genes involved in a particular pathway compared to a number of genes in genelist, BgRatio comparing ratio of genes involved in a pathway to genes in the database, *p*-value, adjusted *p*-value (*p*.adjust), q-value, gene symbol (geneID), and the number of genes from the input list involved in the particular pathway (Count).

## References

[1] Utsunomiya Y.T., Do Carmo A.S., Carvalheiro R., Neves H.H., Matos M.C., Zavarez L.B., Pérez O’Brien A.M., Sölkner J., McEwan J.C., Cole J.B. (2013). Genome-wide association study for birth weight in Nellore cattle points to previously described orthologous genes affecting human and bovine height. BMC Genet..

[2] Pitt D., Sevane N., Nicolazzi E.L., MacHugh D.E., Park S.D.E., Colli L., Martinez R., Bruford M.W., Orozco-terWengel P. (2018). Domestication of cattle: Two or three events?. Evol. Appl..

[3] De Albuquerque L.G. (2006). Proceedings of the 8th World Congress on Genetics Applied to Livestock Production, Belo Horizonte, Brazil, 13–18 August 2006.

[4] Porto-Neto L.R., Reverter A., Prayaga K.C., Chan E.K., Johnston D.J., Hawken R.J., Fordyce G., Garcia J.F., Sonstegard T.S., Bolormaa S. (2014). The genetic architecture of climatic adaptation of tropical cattle. PLoS ONE.

[5] Carvalheiro R., Boison S.A., Neves H.H., Sargolzaei M., Schenkel F.S., Utsunomiya Y.T., O’Brien A.M., Sölkner J., McEwan J.C., Van Tassell C.P. (2014). Accuracy of genotype imputation in Nellore cattle. Genet. Sel. Evol. GSE.

[6] Albuquerque L., Fernandes Júnior G., Carvalheiro R. (2017). Beef Cattle Genomic Selection In Tropical Environments. Proc. Assoc. Advmt. Anim. Breed. Genet..

[7] Fernandes Júnior G.A., Peripolli E., Schmidt P.I., Campos G.S., Mota L.F.M., Mercadante M.E.Z., Baldi F., Carvalheiro R., De Albuquerque L.G. (2022). Current applications and perspectives of genomic selection in Bos indicus (Nellore) cattle. Livest. Sci..

[8] Korte A., Farlow A. (2013). The advantages and limitations of trait analysis with GWAS: A review. Plant Methods.

[9] Mokry F.B., Buzanskas M.E., De Alvarenga Mudadu M., Do Amaral Grossi D., Higa R.H., Ventura R.V., De Lima A.O., Sargolzaei M., Conceição Meirelles S.L., Schenkel F.S. (2014). Linkage disequilibrium and haplotype block structure in a composite beef cattle breed. BMC Genom..

[10] Sanchez M.-P., Tribout T., Kadri N.K., Chitneedi P.K., Maak S., Hozé C., Boussaha M., Croiseau P., Philippe R., Spengeler M. (2023). Sequence-based GWAS meta-analyses for beef production traits. Genet. Sel. Evol..

[11] Irano N., De Camargo G.M.F., Costa R.B., Terakado A.P.N., Magalhães A.F.B., Silva R.M.D.O., Dias M.M., Bignardi A.B., Baldi F., Carvalheiro R. (2016). Genome-Wide Association Study for Indicator Traits of Sexual Precocity in Nellore Cattle. PLoS ONE.

[12] Silva D.O., Fernandes Júnior G.A., Fonseca L.F.S., Mota L.F.M., Bresolin T., Carvalheiro R., De Albuquerque L.G. (2024). Genome-wide association study for stayability at different calvings in Nellore beef cattle. BMC Genom..

[13] Carvalho F.E., Espigolan R., Berton M.P., Neto J.B.S., Silva R.P., Grigoletto L., Silva R.M.O., Ferraz J.B.S., Eler J.P., Aguilar I. (2020). Genome-wide association study and predictive ability for growth traits in Nellore cattle. Livest. Sci..

[14] Reis H.B.D., Carvalho M.E., Espigolan R., Poleti M.D., Ambrizi D.R., Berton M.P., Ferraz J.B.S., De Mattos Oliveira E.C., Eler J.P. (2023). Genome-Wide Association (GWAS) Applied to Carcass and Meat Traits of Nellore Cattle. Metabolites.

[15] Oliveira Júnior G.A., Santos D.J.A., Cesar A.S.M., Boison S.A., Ventura R.V., Perez B.C., Garcia J.F., Ferraz J.B.S., Garrick D.J. (2019). Fine mapping of genomic regions associated with female fertility in Nellore beef cattle based on sequence variants from segregating sires. J. Anim. Sci. Biotechnol..

[16] Börnigen D., Tranchevent L.C., Bonachela-Capdevila F., Devriendt K., De Moor B., De Causmaecker P., Moreau Y. (2012). An unbiased evaluation of gene prioritization tools. Bioinformatics.

[17] Azadifar S., Ahmadi A. (2022). A novel candidate disease gene prioritization method using deep graph convolutional networks and semi-supervised learning. BMC Bioinform..

[18] Fonseca P.A.D.S., Dos Santos F.C., Lam S., Suárez-Vega A., Miglior F., Schenkel F.S., Diniz L.D.A.F., Id-Lahoucine S., Carvalho M.R.S., Cánovas A. (2018). Genetic mechanisms underlying spermatic and testicular traits within and among cattle breeds: Systematic review and prioritization of GWAS results. J. Anim. Sci..

[19] Narayana S.G., de Jong E., Schenkel F.S., Fonseca P.A.S., Chud T.C.S., Powell D., Wachoski-Dark G., Ronksley P.E., Miglior F., Orsel K. (2023). Underlying genetic architecture of resistance to mastitis in dairy cattle: A systematic review and gene prioritization analysis of genome-wide association studies. J. Dairy Sci..

[20] Silva T.D.L., Gondro C., Fonseca P.A.D.S., Da Silva D.A., Vargas G., Neves H.H.D.R., Filho I.C., Teixeira C.D.S., Albuquerque L.G.D., Carvalheiro R. (2023). Testicular hypoplasia in Nellore Cattle: Genetic analysis and functional analysis of genome-wide association study results. J. Anim. Breed. Genet..

[21] Silva T.D.L., Gondro C., Fonseca P.A.D.S., Silva D.A.D., Vargas G., Neves H.H.D.R., Carvalho Filho I., Teixeira C.D.S., Albuquerque L.G.D., Carvalheiro R. (2023). Feet and legs malformation in Nellore cattle: Genetic analysis and prioritization of GWAS results. Front. Genet..

[22] Page M.J., McKenzie J.E., Bossuyt P.M., Boutron I., Hoffmann T.C., Mulrow C.D., Shamseer L., Tetzlaff J.M., Akl E.A., Brennan S.E. (2021). The PRISMA 2020 statement: An updated guideline for reporting systematic reviews. BMJ.

[23] Sarkis-Onofre R., Catalá-López F., Aromataris E., Lockwood C. (2021). How to properly use the PRISMA Statement. Syst. Rev..

[24] Hu Z.-L., Park C.A., Reecy J.M. (2022). Bringing the Animal QTLdb and CorrDB into the future: Meeting new challenges and providing updated services. Nucleic Acids Res..

[25] Sayers E.W., Bolton E.E., Brister J.R., Canese K., Chan J., Comeau D.C., Connor R., Funk K., Kelly C., Kim S. (2022). Database resources of the national center for biotechnology information. Nucleic Acids Res..

[26] Fonseca P.A.S., Suárez-Vega A., Marras G., Cánovas Á. (2020). GALLO: An R package for genomic annotation and integration of multiple data sources in livestock for positional candidate loci. GigaScience.

[27] R Core Team (2016). R: A Language and Environment for Statistical Computing.

[28] Nicolazzi E.L., Caprera A., Nazzicari N., Cozzi P., Strozzi F., Lawley C., Pirani A., Soans C., Brew F., Jorjani H. (2015). SNPchiMp v.3: Integrating and standardizing single nucleotide polymorphism data for livestock species. BMC Genom..

[29] Martin F.J., Amode M.R., Aneja A., Austine-Orimoloye O., Azov A.G., Barnes I., Becker A., Bennett R., Berry A., Bhai J. (2023). Ensembl 2023. Nucleic Acids Res..

[30] Shamimuzzaman M., Le Tourneau J.J., Unni D.R., Diesh C.M., Triant D.A., Walsh A.T., Tayal A., Conant G.C., Hagen D.E., Elsik C.G. (2020). Bovine Genome Database: New annotation tools for a new reference genome. Nucleic Acids Res..

[31] Raney B.J., Barber G.P., Benet-Pagès A., Casper J., Clawson H., Cline M.S., Diekhans M., Fischer C., Navarro Gonzalez J., Hickey G. (2024). The UCSC Genome Browser database: 2024 update. Nucleic Acids Res..

[32] Nelson S.J., Schopen M., Savage A.G., Schulman J.L., Arluk N. (2004). The MeSH translation maintenance system: Structure, interface design, and implementation. Stud. Health Technol. Inform..

[33] Adams W.T., Skopek T.R. (1987). Statistical test for the comparison of samples from mutational spectra. J. Mol. Biol..

[34] Morota G., Peñagaricano F., Petersen J.L., Ciobanu D.C., Tsuyuzaki K., Nikaido I. (2015). An application of MeSH enrichment analysis in livestock. Anim. Genet..

[35] Tsuyuzaki K., Morota G., Ishii M., Nakazato T., Miyazaki S., Nikaido I. (2015). MeSH ORA framework: R/Bioconductor packages to support MeSH over-representation analysis. BMC Bioinform..

[36] Yu G. (2018). Using meshes for MeSH term enrichment and semantic analyses. Bioinformatics.

[37] Chen J., Bardes E.E., Aronow B.J., Jegga A.G. (2009). ToppGene Suite for gene list enrichment analysis and candidate gene prioritization. Nucleic Acids Res..

[38] Guney E., Garcia-Garcia J., Oliva B. (2014). GUILDify: A web server for phenotypic characterization of genes through biological data integration and network-based prioritization algorithms. Bioinformatics.

[39] Aguirre-Plans J., Piñero J., Sanz F., Furlong L.I., Fernandez-Fuentes N., Oliva B., Guney E. (2019). GUILDify v2.0: A Tool to Identify Molecular Networks Underlying Human Diseases, Their Comorbidities and Their Druggable Targets. J. Mol. Biol..

[40] Kominakis A., Hager-Theodorides A.L., Zoidis E., Saridaki A., Antonakos G., Tsiamis G. (2017). Combined GWAS and ‘guilt by association’-based prioritization analysis identifies functional candidate genes for body size in sheep. Genet. Sel. Evol..

[41] Yu G., Wang L.-G., Han Y., He Q.-Y. (2012). clusterProfiler: An R Package for Comparing Biological Themes Among Gene Clusters. OMICS J. Integr. Biol..

[42] Sweett H., Fonseca P.A.S., Suárez-Vega A. (2020). Genome-wide association study to identify genomic regions and positional candidate genes associated with male fertility in beef cattle. Sci Rep.

[43] Warde-Farley D., Donaldson S.L., Comes O., Zuberi K., Badrawi R., Chao P., Morris Q. (2010). The GeneMANIA prediction server: Biological network integration for gene prioritization and predicting gene function. Nucleic Acids Res..

[44] Soares R.A.N., Vargas G., Muniz M.M.M., Soares M.A.M., Cánovas A., Schenkel F., Squires E.J. (2021). Differential gene expression in dairy cows under negative energy balance and ketosis: A systematic review and meta-analysis. J. Dairy Sci..

[45] Oshimori N., Li X., Ohsugi M., Yamamoto T. (2009). Cep72 regulates the localization of key centrosomal proteins and proper bipolar spindle formation. EMBO J..

[46] Alliance of Genome Resources (2022). Tubulin-γ Complex-Associated Protein 2 (TUBGCP2). www.alliancegenome.org/gene/HGNC:18599.

[47] (2022). Alliance of Genome Resources. Espin Like (ESPNL). https://www.alliancegenome.org/gene/HGNC:27937.

[48] Alliance of Genome Resources (2022). Solute Carrier Family 27 Member 1 (SLC27A1). https://www.alliancegenome.org/gene/HGNC:27937.

[49] Alliance of Genome Resources (2022). Acyl-CoA Synthetase Medium Chain Family Member 2A (ACSM2A). https://www.alliancegenome.org/gene/HGNC:32017.

[50] Oshimori N., Ohsugi M., Yamamoto T. (2006). The Plk1 target Kizuna stabilizes mitotic centrosomes to ensure spindle bipolarity. Nat. Cell Biol..

[51] Zhang Z., Liu J., Wu Y., Zhao X., Hao Y., Wang X., Xue C., Wang Y., Zhang R., Zhang X. (2020). Long Noncoding RNA SERTAD2-3 Inhibits Osteosarcoma Proliferation and Migration by Competitively Binding miR-29c. Genet. Test. Mol. Biomark..

[52] Darwish H. (2006). Genomic and Functional Studies of SERTAD3, an Oncogenic Protein of the SERTAD Family of Transcription Factors. Master’s Thesis.

[53] Fu X., Yucer N., Liu S., Li M., Yi P., Mu J.J., Yang T., Chu J., Jung S.Y., O’Malley B.W. (2010). RFWD3-Mdm2 ubiquitin ligase complex positively regulates p53 stability in response to DNA damage. Proc. Natl. Acad. Sci. USA.

[54] Lamsam-Casalotti S., Onoda M., Papadopoulos V., Dym M. (1993). Developmental expression of GTP-binding proteins in rat testes. J. Reprod. Fertil..

[55] Alliance of Genome Resources (2022). Acyl-CoA Synthetase Medium Chain Family Member 4 (ACSM4). https://www.alliancegenome.org/gene/HGNC:32016.

[56] Manna P.R., Stetson C.L., Slominski A.T., Pruitt K. (2016). Role of the steroidogenic acute regulatory protein in health and disease. Endocrine.

[57] Errico A., Vinco S., Ambrosini G., Dalla Pozza E., Marroncelli N., Zampieri N., Dando I. (2023). Mitochondrial Dynamics as Potential Modulators of Hormonal Therapy Effectiveness in Males. Biology.

[58] Alliance of Genome Resources (2022). AlkB Homolog 7 (ALKBH7). https://www.alliancegenome.org/gene/HGNC:21306.

[59] Alliance of Genome Resources (2022). Codanin 1 (CDAN1). https://www.alliancegenome.org/gene/HGNC:1713.

[60] Alliance of Genome Resources (2022). Dual Specificity Tyrosine Phosphorylation Regulated Kinase 2 (DYRK2). https://www.alliancegenome.org/gene/HGNC:3093.

[61] Alliance of Genome Resources (2022). Heat Shock Protein Family A (Hsp70) Member 1A (HSPA1A). https://www.alliancegenome.org/gene/HGNC:5232.

[62] Alliance of Genome Resources (2022). Synaptosome Associated Protein 47 (SNAP47). https://www.alliancegenome.org/gene/HGNC:30669.

[63] Alliance of Genome Resources (2022). ORC Ubiquitin Ligase 1(OBI1). https://www.alliancegenome.org/gene/HGNC:20308.

[64] Nassar J. (2019). Studying the function(s) of OBI1, a novel E3 ubiquitin ligase, involved in DNA replication [Doctoral thesis]. HAL Open Arch..

[65] Alliance of Genome Resources (2022). Transmembrane Protein 120A (TMEM120A). https://www.alliancegenome.org/gene/HGNC:21697.

[66] Batrakou D.G., De Las Heras J.I., Czapiewski R., Mouras R., Schirmer E.C. (2015). TMEM120A and B: Nuclear Envelope Transmembrane Proteins Important for Adipocyte Differentiation. PLoS ONE.

[67] Alliance of Genome Resources (2022). Dual Specificity Phosphatase 29 (DUSP29). https://www.alliancegenome.org/gene/HGNC:23481.

[68] Cooper L.M., West R.C., Hayes C.S., Waddell D.S. (2020). Dual-specificity phosphatase 29 is induced during neurogenic skeletal muscle atrophy and attenuates glucocorticoid receptor activity in muscle cell culture. Am. J. Physiol. Cell Physiol..

[69] Alliance of Genome Resources (2022). Josephin Domain Containing 1 (JOSD1). https://www.alliancegenome.org/gene/HGNC:28953.

[70] Seki T., Gong L., Williams A.J., Sakai N., Todi S.V., Paulson H.L. (2013). JosD1, a membrane-targeted deubiquitinating enzyme, is activated by ubiquitination and regulates membrane dynamics, cell motility, and endocytosis. J. Biol. Chem..

[71] GeneCards LOXL1 Antisense RNA 1 (LOXL1-AS1). https://www.genecards.org/cgi-bin/carddisp.pl?gene=LOXL1-AS1.

[72] Alliance of Genome Resources (2022). Calcium Regulated Heat Stable Protein 1 (*CARHSP1*). https://www.alliancegenome.org/gene/HGNC:17150.

[73] Kociucka B., Stachecka J., Szydlowski M., Szczerbal I. (2017). Rapid Communication: The correlation between histone modifications and expression of key genes involved in accumulation of adipose tissue in the pig. J. Anim. Sci..

[74] Alliance of Genome Resources (2022). Translocase of Inner Mitochondrial Membrane 8A (TIMM8A). https://www.alliancegenome.org/gene/HGNC:11817.

[75] Alliance of Genome Resources (2022). Terminal Nucleotidyltransferase 4A (TENT4A). https://www.alliancegenome.org/gene/HGNC:16705.

[76] Alliance of Genome Resources (2022). Ankyrin Repeat Domain 34B (ANKRD34B). https://www.alliancegenome.org/gene/HGNC:33736.

[77] Tartaglia G.G. (2016). The Grand Challenge of Characterizing Ribonucleoprotein Networks. Front. Mol. Biosci..

[78] Zhang J. (2023). Patterns and evolutionary consequences of pleiotropy. Annu. Rev. Ecol. Evol. Syst..

[79] Alliance of Genome Resources (2022). Ring Finger and WD Repeat Domain 3 (RFWD3). https://www.alliancegenome.org/gene/HGNC:25539.

[80] Ménézo Y., Dale B., Cohen M. (2010). DNA damage and repair in human oocytes and embryos: A review. Zygote.

[81] Inano S., Sato K., Katsuki Y., Kobayashi W., Tanaka H., Nakajima K., Nakada S., Miyoshi H., Knies K., Takaori-Kondo A. (2017). RFWD3-Mediated Ubiquitination Promotes Timely Removal of Both RPA and RAD51 from DNA Damage Sites to Facilitate Homologous Recombination. Mol. Cell.

[82] Alvarenga A.B., Oliveira H.R., Chen S.Y., Miller S.P., Marchant-Forde J.N., Grigoletto L., Brito L.F. (2021). A Systematic Review of Genomic Regions and Candidate Genes Underlying Behavioral Traits in Farmed Mammals and Their Link with Human Disorders. Anim. Open Access J..

[83] Elsik C.G., Tellam R.L., Worley K.C., Gibbs R.A., Muzny D.M., Weinstock G.M., Adelson D.L., Eichler E.E., Elnitski L., Bovine Genome Sequencing and Analysis Consortium (2009). The genome sequence of taurine cattle: A window to ruminant biology and evolution. Science.

[84] Weitzman J.B. (2000). Comparing cows with humans. Genome Biol..

